# Association between early hyponatremia and neurological diagnoses in infants with perinatal asphyxia

**DOI:** 10.1007/s00431-026-07325-3

**Published:** 2026-08-11

**Authors:** Niina Viitaharju, Kjell Helenius, Eliisa Löyttyniemi, Bishwesvar Singh, Vilhelmiina Parikka

**Affiliations:** 1 Department of Clinical Medicine, University of Turku, Turku, Finland; 2 Department of Pediatrics and Adolescent Medicine, Turku University Hospital, Turku, Finland; 3 Department of Biostatistics, University of Turku and Turku University Hospital, Turku, Finland; 4 MediCity Research Laboratories, University of Turku, Turku, Finland; 5 InFLAMES Research Flagship Center, University of Turku, Turku, Finland

**Keywords:** Hypoxic-ischemic encephalopathy, Neonate, Hyponatremia, Hearing disorders, Cerebral palsy, Epilepsy

## Abstract

To investigate the association between early hyponatremia and long-term neurodevelopmental diagnoses in infants with perinatal asphyxia. This retrospective study included infants born at ≥ 36 weeks of gestation with an ICD-10 diagnosis of perinatal asphyxia and/or hypoxic-ischemic encephalopathy. Data on sodium levels within the first 72 h of life, early postnatal weight change, and clinical follow-up were extracted from medical records. The outcome was death or any ICD-10 diagnoses indicating a neurodevelopmental disorder (NDD), including epileptic, paralytic, hearing, visual and psychiatric/developmental disorders. For multivariable analysis, estimates were adjusted for sex, mode of delivery, and therapeutic hypothermia. Among 269 neonates, 193 (71.7%) had at least one sodium value < 135 mmol/l, 71 (26.4%) < 130 mmol/l and 12 (4.5%) < 125 mmol/l during the first 72 h of life. The median follow-up time was 11.2 years (range 2.4 – 14.8). NDD or death occurred in 57 infants (21.2%). In multivariable analyses, sodium < 125 mmol/l was significantly associated with NDD or death (aOR = 3.85, 95% Cl 1.04—14.22). Sodium < 130 mmol/l was significantly associated with cerebral palsy (aOR = 8.94, 95% Cl 2.24—35.67), epilepsy (aOR = 20.75, 95% Cl 2.39—180.23) and hearing disorders (aOR = 6.09, 95% CI 1.32—28.18).

*Conclusions*: Our study shows that hyponatremia during the first 72 h of life in infants with perinatal asphyxia is associated with an increased risk of later neurodevelopmental disorders. It remains unclear whether hyponatremia contributes causally to adverse outcomes or primarily serves as a biomarker of severe injury.

**Table:** 

**What is Known:** • *Perinatal asphyxia may lead to long-term neurodevelopmental disorders, including cognitive impairment, epilepsy, cerebral palsy, and sensory impairments.* • *Hyponatremia is common in infants with birth asphyxia and has been associated with low Apgar scores and the severity of hypoxic-ischemic encephalopathy.*
**What is New:** • *In this study, ICD-10 diagnoses of neurological disorders following perinatal asphyxia were examined using an extended follow-up period.* • *Early hyponatremia was significantly associated with cerebral palsy, epilepsy and hearing disorders.*

**Supplementary Information:**

The online version contains supplementary material available at https://doi.org/10.1007/s00431-026-07325-3.

## Introduction

Perinatal asphyxia in infants results from insufficient perfusion and/or oxygen deprivation around the time of birth. Although there is no universally accepted definition, low Apgar scores and hypoxia-induced metabolic acidosis are commonly used indicators. Some infants with perinatal asphyxia develop hypoxic-ischemic encephalopathy (HIE), which may lead to long-term neurodevelopmental disabilities such as cognitive impairment, epilepsy, cerebral palsy or death [1, 2]. Hypoxic injury can also result in multiorgan failure, including acute kidney injury. Therapeutic hypothermia is the only evidence-based treatment for neonatal HIE [3]. It has been shown to reduce mortality and the risk of severe neurological impairment in infants with moderate to severe HIE [4]. However, despite treatment, a significant proportion of survivors still experience long-term disabilities, and early identification of infants who may benefit from intervention remains a clinical challenge.

Numerous studies have investigated predictors of short- and long-term outcomes in infants with perinatal asphyxia, including brain MRI imaging, electroencephalography, Apgar scores and biomarkers [5–8]. Laboratory parameters are a key area of ongoing research. Although low umbilical artery pH has been associated with HIE [9], both low umbilical pH and base excess are poor predictors of the long-term neurological outcome [10].

Perinatal asphyxia can cause end-organ damage, including acute kidney injury and neuroendocrine disturbances, such as syndrome of inappropriate secretion of antidiuretic hormone (SIADH). Both conditions can lead to fluid overload and subsequent hyponatremia. Hyponatremia may also be iatrogenic, resulting from fluid overload or inadequate fluid restriction during intensive care. Infants with perinatal asphyxia have significantly lower sodium levels compared to control groups [11–15], and hyponatremia is a common finding in these patients [15, 16].

Previous studies have reported an association between sodium levels and the severity of perinatal asphyxia [17, 18] or HIE [16, 19]. A few studies have also suggested an association between hyponatremia and low Apgar scores [11, 12, 14]. One study reported an association between hyponatremia and both mortality and prolonged hospital stay [20]. In addition, a retrospective cohort study of infants with moderate to severe HIE suggested that therapeutic hypothermia may contribute to fluid balance disturbances and the incidence of hyponatremia. The incidence of hyponatremia was higher among infants who received therapeutic hypothermia (76%) compared to those who did not (48%) [21].

There is a limited number of studies on hyponatremia following perinatal asphyxia, and the available studies have had short follow-up periods. The aim of this study was to investigate the prevalence of early hyponatremia and its prognostic value for long-term neurological outcomes in infants with perinatal asphyxia using a longer follow-up compared to previous studies.

## Methods

### Study population

Infants born at ≥ 36 weeks of gestation between January 1, 2010, and June 1, 2022, within the Turku University Hospital catchment area and admitted to neonatal intensive care for perinatal asphyxia (ICD-10 category P21) or HIE (ICD-10 subcategory P91.0) were retrospectively identified from medical records. Infants were excluded if no laboratory data were available for analysis or if follow-up occurred outside our hospital catchment area, resulting in a lack of outcome data. Also, infants with severe congenital anomalies, congenital viral infections, or those later diagnosed with genetic, metabolic, or neuromuscular conditions associated with neurodevelopmental diagnoses were excluded.

Since 2010, our hospital has followed a clinical protocol for therapeutic hypothermia, involving cooling to 33.5 °C for 72 h. Infants with moderate to severe HIE (Sarnat stages 2–3), but not those with mild HIE (Sarnat stage 1), are eligible for therapeutic hypothermia.

### Sodium and weight

All sodium values recorded within the first 72 h of life were collected from the electronic medical records. Analyses focused on infants’ lowest recorded sodium values. Hyponatremia was defined as a sodium concentration < 135 mmol/l. Additional analyses were performed using alternative thresholds of < 130 mmol/l and < 125 mmol/l. Sodium measurements were acquired as part of routine care either as part of blood gas analysis (ABL Radiometer, Brønshøj, Denmark) or through standard laboratory sampling (heparinized plasma, indirect ion selective electrode technique).

Infants’ birth weights and all recorded weights during the first five days of life were collected. For each infant, the highest measured weight during this period, excluding the birthweight, was identified. The difference between this value and the birth weight was defined as early weight change and was used to assess its association with hyponatremia.

### Outcome

The outcome was defined as any occurrence of death or an ICD-10 diagnosis indicating a neurodevelopmental disorder (NDD) during follow-up. The diagnostic categories included F70-98 (psychiatric or developmental disorders), G40-41 (epileptic disorders), G80-83 (cerebral palsy and other paralytic syndromes), H47-49 and H53-54 (visual disorders) or H90-91 (hearing disorders). Diagnostic categories were also analyzed individually. All diagnoses were made within pediatric specialized health care in our tertiary university hospital, involving specialists such as pediatricians, pediatric neurologists, ophthalmologists, otorhinolaryngologists, audiologists and pediatric psychiatrists.

### Statistical methods

Continuous variables are summarized using the median together with lower (Q1) and upper (Q3) quartiles, or range, as appropriate. Categorical variables are reported as counts and percentages.

Logistic regression analyses (both univariate and multivariable) were performed, and results are reported as odds ratios (ORs) along with 95% confidence intervals (CIs), and p-values. Multivariable logistic regression models were constructed for primary and secondary outcomes including four explanatory variables: sex, mode of delivery, therapeutic hypothermia and derived variable for minimum sodium value (< 125 mmol/l, < 130 mmol/l, or < 135 mmol/l).

The association between early weight change over 5 days after birth and categorised minimum sodium values was analysed using two-way analysis of variance, with therapeutic hypothermia included as a second explanatory variable in the model.

*P*-values less than 0.05 (two-tailed) were considered statistically significant. The data analyses were generated using SAS software, Version 9.4 of the SAS System for Windows (SAS Institute Inc., Cary, NC, USA).

## Results

A total of 304 infants met the inclusion criteria, of whom 35 were excluded due to lack of follow-up data, missing sodium values, or severe congenital anomalies, leaving a total of 269 eligible infants. Infants’ background information is shown in Table 1 and clinical care details in supplementary Table 1. Of the 269 infants, 172 (63.9%) were delivered vaginally and 97 (36.1%) by cesarean section. The median gestational age was 40.3 weeks, and the mean birth weight was 3575 g (SD 540). A total of 156 (58.0%) infants were male and 113 (42.0%) female. The median Apgar scores were 3 at 1 min and 6 at 5 min. The mean umbilical arterial pH was 7.07 (SD 0.14). Fourty-four (16.4%) infants received therapeutic hypothermia.

**Table 1:** Distribution of background variables among 269 infants with the diagnosis of birth asphyxia (ICD-10 category P21) and/or hypoxic-ischemic encephalopathy (ICD-10 subcategory P91.0)

	Total (*n* = 269)	Therapeutic hypothermia (*n* = 44)	No therapeutic hypothermia (*n* = 225)
**Gestational age, weeks**	40.1 (39.3, 41.3)	40.1 (39.4, 41.3)	40.2 (39.3, 41.3)
**Birth weight, grams** (*n* = 268)	3575 (540)	3655 (527)	3559 (542)
**Sex**			
Male	156 (58.0%)	26 (59.1%)	130 (57.8%)
Female	113 (42.0%)	18 (40.9%)	95 (42.2%)
**Multiple gestation**	4 (1.5%)	1 (2.3%)	3 (1.3%)
**Apgar scores**			
5 min (*n* = 264)	6 (4, 6)	3 (1, 4)	6 (4, 7)
**Umbilical arterial pH** (*n* = 241)	7.07 (0.14)	7.06 (0.16)	7.07 (0.13)
**Umbilical vein pH** (*n* = 221)	7.19 (0.15)	7.19 (0.14)	7.18 (0.17)
**Mode of delivery**			
Vaginal delivery	172 (63.9%)	27 (61.4%)	145 (64.4%)
Cesarean section	97 (36.1%)	17 (38.6%)	80 (35.6%)
**Delivery room management**			
Mask ventilation	208 (77.6%)	40 (90.9%)	168 (75.0%)
Intubation	48 (17.9%)	28 (63.6%)	20 (8.9%)
Chest compression	22 (8.2%)	15 (34.1%)	7 (3.1%)
Adrenaline	9 (3.4%)	9 (20.5%)	0 (0%)
Other resuscitation medications	2 (0.8%)	2 (4.6%)	0 (0%)
IV fluid bolus	41 (15.3%)	19 (43.2%)	22 (9.8%)
Blood/Red blood cell transfusion	2 (0.8%)	1 (2.3%)	1 (0.5%)
Time to first spontaneous breath (min) (*n* = 247)	11.7 (10.6)	21.6 (11.4)	9.8 (9.3)
Time to spontaneous circulation (min) (*n* = 251)	3.2 (3.9)	7.4 (7.1)	2.3 (2.0)
**Age at hospital discharge, days**	6 (4,8)	12 (8, 17)	6 (4, 7)

^1^Values are presented as n (%), mean (SD) or median (IQR), as appropriate, unless otherwise stated

### Sodium values at different time points

**Fig. 1:**
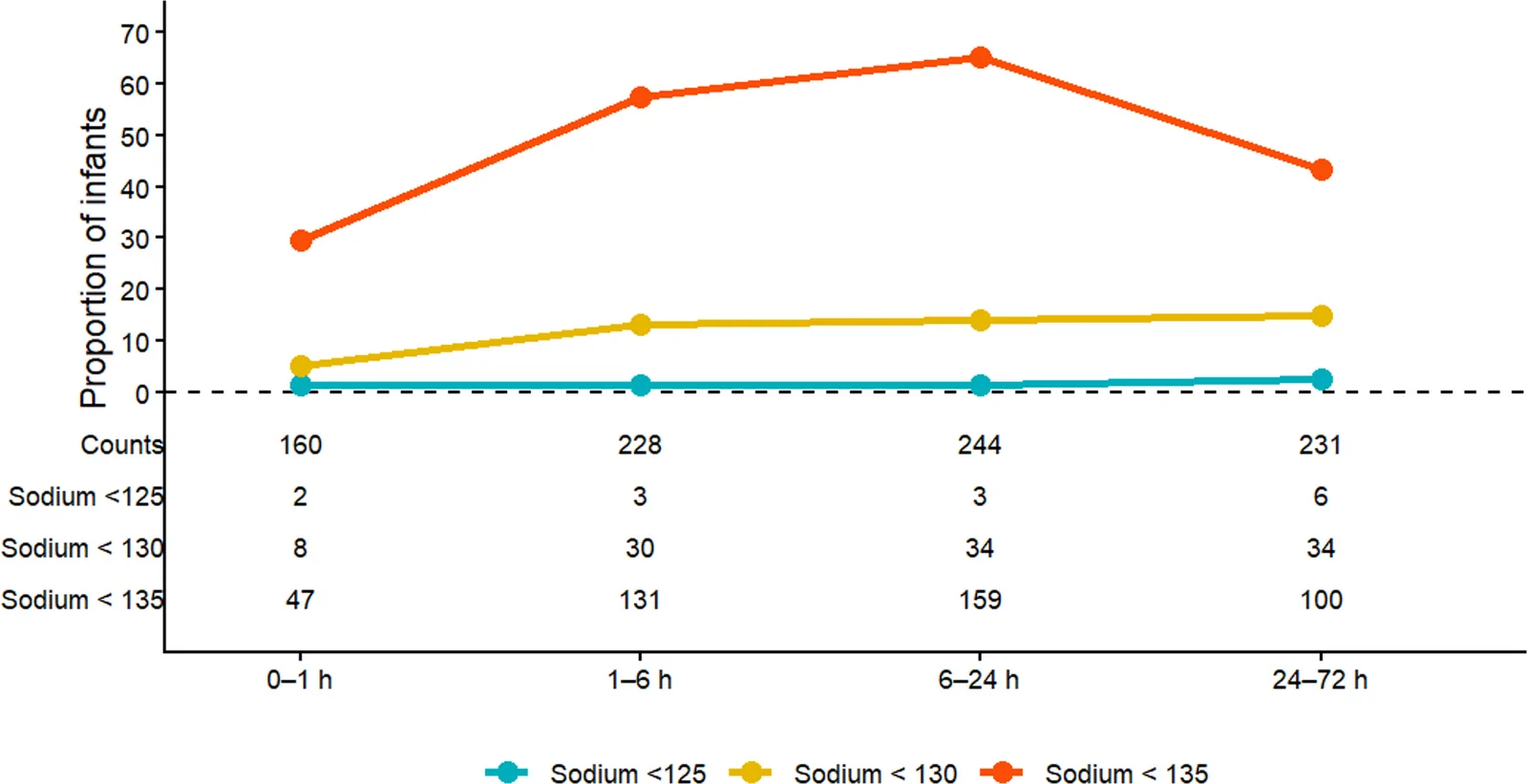
The relative frequency of hyponatremia across different time intervals during the first 72 h

During the first 72 h of life, 12 infants (4.5%) had at least one measured sodium value < 125 mmol/l, 71 infants (26.4%) had at least one sodium value < 130 mmol/l, and 193 infants (71.7%) had at least one sodium value < 135 mmol/l. Figure 1 presents more detailed data of sodium values.

### Follow-up and neurodevelopmental disorders

**Fig. 2:**
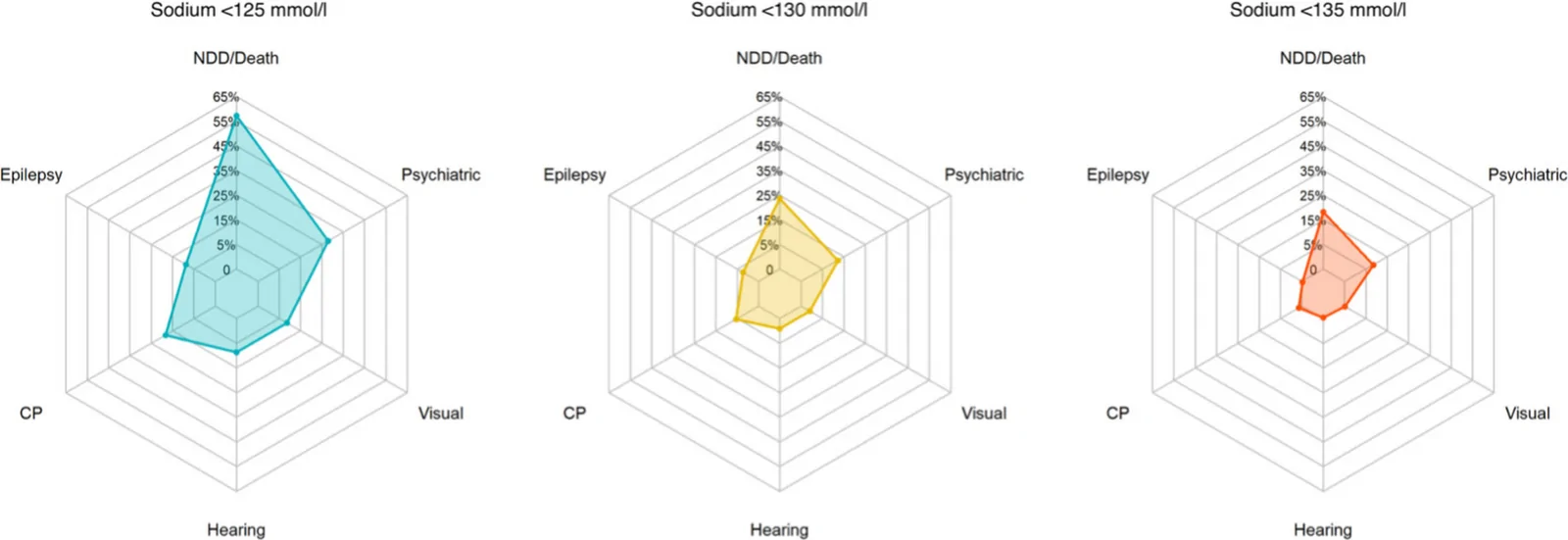
Incidence of neurodevelopmental disorders across different hyponatremia categories (Na < 125 mmol/l, < 130 mmol/l, < 135 mmol/l)

The median follow-up time was 11.2 years (range 2.4–14.8 years). Of the 269 infants, 52 (19.3%) were diagnosed with at least one of the studied neurodevelopmental disorders, and 5 infants (1.9%) died during the first two weeks of life. Among the 52 infants with neurodevelopmental disorders, 13 (4.8%) were diagnosed with cerebral palsy, 9 (3.3%) with epileptic disorders, 9 (3.3%) with hearing disorders, 12 (4.5%) with visual disorders and 38 (14.1%) with psychiatric/developmental disorders. Figure 2 shows the distribution of diagnoses in different sodium categories.

### Hyponatremia and neurological outcome

**Fig. 3:**
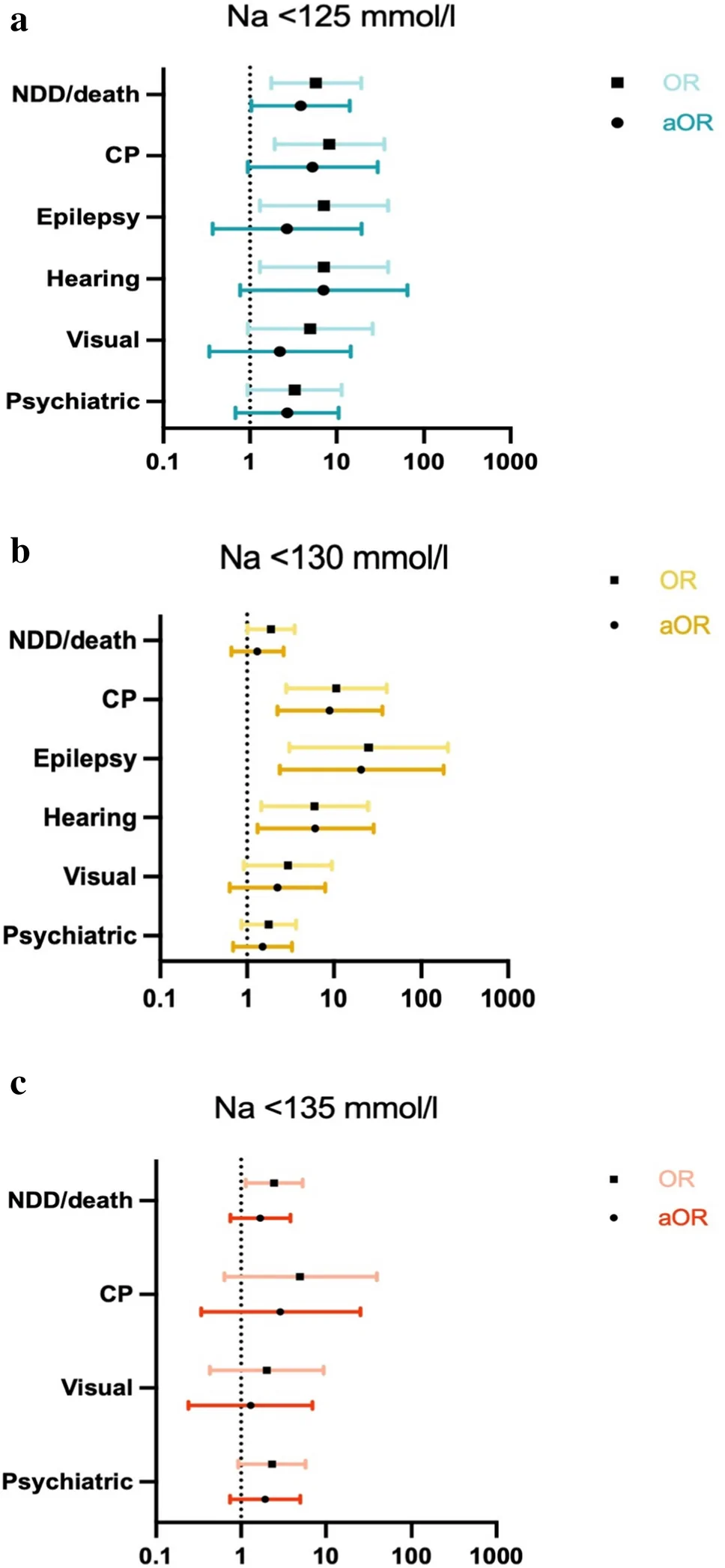
**a**-**c**. Forest plot of the unadjusted odds ratios (OR) and adjusted odds ratios (aOR) for the associations between hyponatremia (a. < 125 mmol/l, b. < 130 mmol/l, c. < 135 mmol/l) and neurodevelopmental diagnoses

Infants with hyponatremia were more likely to be diagnosed with neurodevelopmental disorders (Fig. 3a-c, Table 2). In univariate analyses, sodium < 125 mmol/l was significantly associated with NDD or death (OR = 5.79, 95% CI 1.77–19.02), cerebral palsy (OR = 8.23, 95% CI 1.93–35.15), epilepsy (OR = 7.14, 95% CI 1.31–38.86) and hearing disorders (OR = 7.14, 95% CI, 1.31–38.86). In the multivariable analyses (Fig. 3a-c, Table 2), the association remained statistically significant for NDD or death (aOR = 3.85, 95% CI 1.04–14.22). There were no statistically significant associations between any level of hyponatremia and visual disorders and psychiatric/developmental disorders.

**Table 2:** Univariate and multivariable associations between hyponatremia (sodium < 125 mmol/l, < 130 mmol/l and < 135 mmol/l) within the first 72 h of life and ICD-10 diagnoses indicating neurodevelopmental disorders in infants with perinatal asphyxia

Outcomes	Min sodium	n (%) with outcome	OR (95%Cl)	aOR (95%Cl)^1^
NDD/death (*n* = 57)	< 125 mmol/l	7 (58.3%)	**5.79 (1.77, 19.02)**	**3.85 (1.04, 14.22)**
	< 130 mmol/l	21 (29.6%)	**1.89 (1.01, 3.53)**	1.31 (0.66, 2.63)
	< 135 mmol/l	48 (24.9%)	**2.46 (1.14, 5.32)**	1.69 (0.75, 3.83)
CP (*n* = 13)	< 125 mmol/l	3 (25.0%)	**8.23 (1.93, 35.15)**	5.29 (0.95, 29.50)
	< 130 mmol/l	10 (14.1%)	**10.66 (2.84, 39.96)**	**8.94 (2.24, 35.67)**
	< 135 mmol/l	12 (6.2%)	4.97 (0.64, 38.92)	2.91 (0.34, 24.94)
Epilepsy (*n* = 9)	< 125 mmol/l	2 (16.7%)	**7.14 (1.31, 38.86)**	2.67 (0.37, 19.11)
	< 130 mmol/l	8 (11.3%)	**25.02 (3.07, 203.90)**	**20.75 (2.39, 180.23)**
	< 135 mmol/l	9 (4.7%)	NA	NA
Hearing disorders (*n* = 9)	< 125 mmol/l	2 (16.7%)	**7.14 (1.31, 38.86)**	7.07 (0.77, 64.63)
	< 130 mmol/l	6 (8.5%)	**6.00 (1.46, 24.68)**	**6.09 (1.32, 28.18)**
	< 135 mmol/l	9 (4.7%)	NA	NA
Visual disorders (*n* = 12)	< 125 mmol/l	2 (16.7%)	4.94 (0.95, 25.58)	2.22 (0.34, 14.57)
	< 130 mmol/l	6 (8.5%)	2.96 (0.92, 9.48)	2.24 (0.63, 7.95)
	< 135 mmol/l	10 (5.2%)	2.02 (0.43, 9.45)	1.30 (0.24, 6.96)
Psychiatric disorders (*n* = 38)	< 125 mmol/l	4 (33.3%)	3.28 (0.94, 11.49)	2.69 (0.68, 10.61)
	< 130 mmol/l	14 (19.7%)	1.78 (0.86, 3.67)	1.51 (0.69, 3.28)
	< 135 mmol/l	32 (16.6%)	2.32 (0.93, 5.80)	1.93 (0.74, 5.02)

^1^Adjusted for sex, mode of delivery and therapeutic hypothermia

In univariate analyses, there was a statistically significant association between sodium < 130 mmol/l and NDD or death (OR = 1.89, 95% CI 1.01–3.53), cerebral palsy (OR = 10.66, 95% CI 2.84–39.96), epilepsy (OR = 25.02, 95% CI 3.07–203.90), and hearing disorders (OR = 6.00, 95% CI 1.46–24.68). In the multivariable analyses the association remained statistically significant for cerebral palsy (aOR = 8.94, 95% CI 2.24–35.67), epilepsy (aOR = 20.75, 95% CI 2.39–180.23), and hearing disorders (aOR = 6.09, 95% CI 1.32–28.18).

In univariate analyses, sodium < 135 mmol/l was significantly associated with NDD or death (OR = 2.46, 95% CI 1.14–5.32). In multivariable analyses, no significant associations were observed for any of the diagnoses.

### Hyponatremia and weight

Of the 269 infants, 241 had a recorded birth weight and at least one weight measurement during the first five days of life. Among these infants, the mean difference between the highest recorded weight during the first five days and the birth weight was + 36 g (SD 162). The weight change was greater in infants who received therapeutic hypothermia (+ 246 g, SD 182) than in those who were not treated with therapeutic hypothermia (- 1 g, SD 126.4).

**Fig. 4:**
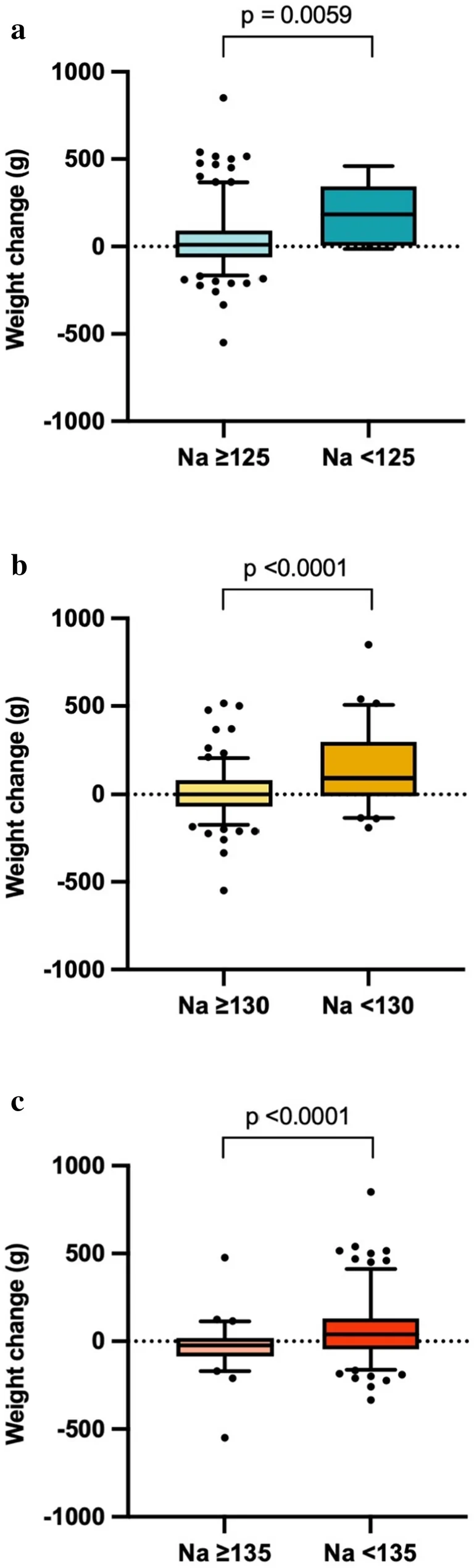
**a**-**c**. Weight change during the first five days of life, expressed as the difference between the highest measured weight and the birth weight, across different hyponatremia categories (a. < 125 mmol/l, b. < 130 mmol/l, c. < 135 mmol/l)

A similar pattern was observed when maximum weight change was compared between infants with at least one low sodium value and those without. The mean difference between the highest recorded weight and the birth weight was greater among infants with at least one sodium value < 125 mmol/l (+ 184.6 g, SD 53.6) than among those without (+ 32.6 g, SD 10.6; *p* = 0.0059; Fig. 4a). However, this association disappeared after adjustment for therapeutic hypothermia (*p* = 0.64). A similar association was observed for infants with at least one sodium value < 130 mmol/l (127.1 g, SD 19.5) compared with those without (6.39 g, SD 11.7; *p* < 0.0001; Fig. 4b). The association remained significant after adjustment for therapeutic hypothermia (*p* = 0.0149). Weight change was also greater in infants with at least one sodium value < 135 mmol/l than in those without (*p* = 0.0324; Fig. 4c).

## Discussion

We found a statistically significant association between early severe hyponatremia and NDD or death. Analyses of individual diagnostic categories revealed significant associations between sodium < 130 mmol/l and cerebral palsy, epilepsy, and hearing disorders. There were no significant associations between mild hyponatremia (Na < 135 mmol/l) and neurological outcomes.

To our knowledge, no previous studies have investigated the association between hyponatremia and long-term outcomes in infants with perinatal asphyxia. However, earlier research has explored the relationship between hyponatremia and the severity of birth asphyxia, supporting the plausibility of our findings. Basu et al. conducted a prospective study comparing infants with perinatal asphyxia to a control group without asphyxia. They found that mean sodium levels immediately after birth were significantly lower in asphyxiated infants compared to controls, and that lower sodium values were associated with lower Apgar scores [14]. Similarly, Bhoye et al. and Acharya et al. reported associations between hyponatremia and the stage of HIE [16, 19]. Both studies demonstrated that decreasing serum sodium levels were associated with increasing severity of HIE. Bhoye et al. also observed a progressive decline in sodium levels over time in infants with stage 2 and 3 HIE. Also, a retrospective study by Thakur et al. involving 88 infants with perinatal asphyxia found a direct association between hyponatremia and the severity of perinatal asphyxia [17].

In asphyxiated infants, hyponatremia may result from several factors, including acute kidney injury (AKI), syndrome of inappropriate antidiuretic hormone secretion (SIADH), or excessive fluid intake. Perinatal asphyxia is one of the main risk factors of AKI. In a meta-analysis of 201 studies from 45 countries, the incidence of any AKI in infants with perinatal asphyxia was 34%, while the incidence of severe AKI was 18% [22]. Bozkurt et al. investigated the incidence and severity of AKI in infants with perinatal asphyxia treated with therapeutic hypothermia and reported an AKI incidence of 29.5%, indicating that AKI remains common despite the use of therapeutic hypothermia [23]. The role of therapeutic hypothermia as a risk factor for electrolyte disturbances has been discussed as well. The effect of therapeutic hypothermia on electrolyte balance and weight was studied in a retrospective cohort by Prempunpong et al. involving 67 infants with moderate or severe HIE. Their data showed that infants who received hypothermia had greater weight gain and higher incidence of hyponatremia than the control group [21]. In our study, hyponatremia was associated with excessive weight gain during the first days of life, suggesting a dilutional origin of hyponatremia.

Sodium, as a major extracellular ion, plays a crucial role in regulating osmotic balance. A decrease in extracellular osmolality due to hyponatremia causes water to move into brain cells to equalize the osmotic difference, which can disrupt cellular homeostasis and lead to cellular swelling and cerebral edema. Although hyponatremia is a well-recognised complication in older patients, less attention has been paid to its occurrence and significance in infants. It is tempting to hypothesize that preventing excessive weight gain and/or hyponatremia might help reduce brain swelling and thereby improve outcomes. However, further studies are needed to clarify to what extent hyponatremia acts as a marker of severe condition versus a direct contributor to unfavorable outcomes. Some previous studies have evaluated the effects of fluid restriction in infants with HIE. In these studies, restricted fluid intake could not reduce mortality, neurodevelopmental disabilities or the incidence of abnormalities in brain MRI [24–26]. In one of the studies, sodium intake was also restricted in addition to total fluid intake, which resulted in significantly lower serum sodium concentrations and an increased prevalence of hyponatremia.

Previous studies have shown a correlation between sodium levels and the severity of perinatal asphyxia or HIE. However, to our knowledge, associations between hyponatremia and long-term neurodevelopmental outcomes have not been investigated. Previous studies were conducted on relatively small cohorts, with a maximum of about 150 infants, and sodium measurements were typically obtained only immediately after birth or at predefined time points. In our study, we aimed to assemble as comprehensive a dataset as possible, including asphyxiated infants with and without HIE, and incorporating all sodium values measured during the first 72 h. We believe that the use of real-word clinical data provides new insights, although it can be regarded both as a strength and a limitation of the study.

In this retrospective study involving 269 infants, the median follow-up time was 11 years. While the extended follow-up period represents a significant strength of our study, the non-standardized follow-up duration varying from 2.4 years to 14.8 years can also be considered a limitation. Another challenge was the variable number of sodium measurements per infant. Due to this limitation, we chose to analyze the minimum measured sodium in each infant instead of e.g. the duration of hyponatremia episodes through serial measurements. We also presented analyses on the change of infants’ weight during the first five days but have no other detailed documentation on fluid balance. More comprehensive data on fluid intake and output could have provided valuable insights into the underlying causes of hyponatremia.

While HIE severity is a known risk factor for adverse neurological outcomes, its effect needs to be taken into account when assessing outcomes. A major limitation of our study is the lack of access to the exact clinical criteria used to assess HIE severity. Instead, we used receipt of therapeutic hypothermia as a proxy for Sarnat stage 2–3 HIE, since our unit’s protocol from 2010 onwards requires stage 2 or 3 criteria for eligibility.

Although our study included 269 infants, the assessed outcomes were relatively rare, leading to unstable estimates as displayed by the wide confidence intervals. Consequently, associations should be interpreted with caution, and no causal relationships can be inferred. Nevertheless, our findings are intriguing and raise several important questions relating to fluid and electrolyte imbalance and their potential impact on neurodevelopmental outcomes.

## Conclusions

Hyponatremia during the first 72 h of life was associated with neurodevelopmental disorders. Even sodium values below 130 mmol/l were significantly associated with hearing disorders, epilepsy, and cerebral palsy. However, it remains unclear whether hyponatremia directly contributes to poor outcomes or primarily serves as a biomarker of severe injury. Further research is needed to clarify the role of early hyponatremia in relation to neurodevelopmental disorders, as well as to better understand its underlying causes.

## Supplementary Information

Below is the link to the electronic supplementary material.

Supplementary file1 (DOCX 14.6 KB)

## Data Availability

No datasets were generated or analysed during the current study.

## Abbreviations

AKI: Acute kidney injury
CP: Cerebral palsy
HIE: Hypoxic-ischemic encephalopathy
MRI: Magnetic resonance imaging
NDD: Neurodevelopmental disorder
SIADH: Syndrome of inappropriate antidiuretic hormone secretion
