# Supplementary material for: Association between early hyponatremia and neurological diagnoses in infants with perinatal asphyxia
Source: Eur J Pediatr. 2026 Aug 11;185(9):656. doi: 10.1007/s00431-026-07325-3 (PMC13461789; doi:10.1007/s00431-026-07325-3)
Supplement: Supplementary file 1 [file 431_2026_7325_MOESM1_ESM.docx]

**Supplementary table 1.** Clinical care detail among 269 infants with the diagnosis of birth asphyxia (ICD-10 category P21) and/or hypoxic-ischemic encephalopathy (ICD-10 subcategory P91.0).

|  | **Total**  **(n=269)** | **Therapeutic hypothermia (n = 44)** | **No therapeutic hypothermia**  **(n = 225)** |
| --- | --- | --- | --- |
| **Clinical care**  Mechanical ventilation  Medications  Sedative  Inotropic  Anticonvulsant | 74 (27.5%)  90 (33.5%)  36 (13.4%)  30 (11.2%) | 39 (88.6%)  40 (90.9%)  12 (27.3%)  19 (44.2%) | 35 (15.6%)  50 (22.2%)  24 (10.7%)  11 (4.9%) |
